# Polyomavirus-Specific Cellular Immunity: From BK-Virus-Specific Cellular Immunity to BK-Virus-Associated Nephropathy?

**DOI:** 10.3389/fimmu.2015.00307

**Published:** 2015-06-16

**Authors:** Manon Dekeyser, Hélène François, Séverine Beaudreuil, Antoine Durrbach

**Affiliations:** ^1^Nephrology Department, IFRNT, Bicêtre Hospital, Le Kremlin Bicêtre, France; ^2^UMRS1197, INSERM, Villejuif, France; ^3^University Paris South, Orsay, France

**Keywords:** polyomavirus, BK-virus, renal transplantation, anti-viral immunity, polyfunctionality

## Abstract

In renal transplantation, BK-virus (BKV)-associated nephropathy has emerged as a major complication, with a prevalence of 1–10% and graft loss in >50% of cases. BKV is a member of the polyomavirus family and rarely induces apparent clinical disease in the general population. However, replication of polyomaviruses, associated with significant organ disease, is observed in patients with acquired immunosuppression. Monitoring of specific immunity combined with viral load could be used to individually assess the risk of viral reactivation and virus control. We review the current knowledge on BKV-specific cellular immunity and, more specifically, in immunocompromised patients. In the future, immune-based therapies could allow us to treat and prevent BKV-associated nephropathy.

## Introduction

BK-virus (BKV) is a human polyomavirus, first isolated in 1971, which belongs to a subfamily of papovaviridae, and includes two main strains associated with human disease: BKV and JC-virus (JCV). BKV is frequently responsible for irrelevant infection in the general population. Up to 85% of adults have been exposed to the virus (as determined by serological testing), but recurrence of the virus can be observed with immunosuppression, suggesting the presence of an asymptomatic, latent infection. Except for children receiving a bone-marrow transplant, in most cases, in adults, BKV infection corresponds to recurrence of the virus rather than a *de novo* infection after renal transplantation. Human BKV has been associated with interstitial nephritis and ureteral stenosis in the renal transplant, and hemorrhagic cystitis in recipients of a bone-marrow transplant. BKV nephropathy, associated with viral recurrence, occurs in 1–10% of renal transplants and has emerged as a major complication after renal transplantation, often leading to graft loss (1). The absence of a non-nephrotoxic antiviral therapy leads to decrease immunosuppression to control viral replication, but can lead to organ rejection. The role of the immune system in controlling this virus is still poorly understood.

## Incidence and Clinical Manifestations

### Polyomaviruses in the general population

Polyomaviruses are widespread in the general population but rarely induce apparent clinical disease or pathology. This virus family coevolved with their hosts, as evidenced by their high prevalence and low morbidity (2, 3). Currently, the human polyomavirus family consists of 10 members. The most common are polyomavirus hominis 1 and 2, better known as BKV and JCV, respectively, named after the initials of the patients from whom they were first isolated in the 1970s (3). With a seroprevalence of more than 80% for BKV and 50% for JCV, epidemiological studies in the general population indicate early and high exposure to these polyomaviruses (4, 5). The primary infection is often subclinical and without specific symptoms. Natural transmission has not been fully clarified but likely occurs via the respiratory or oral route. After a primary infection, BKV and JCV are known to persist in the reno-urinary tract but mechanisms involved for the location of the virus in this epithelial remain unknown (3). Asymptomatic urinary shedding of BKV and JCV has been observed in 7 and 19% of healthy blood donors aged 20–59 years, respectively, with median viral loads of 3.51 and 4.64 log copies/mL, respectively. Neither BKV nor JCV viremia has been detected in plasma in immunocompetent individuals (4).

### Polyomaviruses in immunocompromised patients

Polyomaviruses are latent viruses that can replicate in patients with acquired immunodeficiency. Significant organ diseases associated with the replication of polyomaviruses have been observed in patients with chronic acquired immunosuppression caused by organ transplantation treatments, human immunodeficiency virus (HIV) infection, or multiple sclerosis treated with natalizumab. BKV and JCV display similar behavior, with high prevalence in the general population and related diseases occurring almost only in immunocompromised population. Similar mechanisms for the replication control for these two viruses are required, implicating the host response. However, BKV disease does not directly correlate with the level of immunosuppression but usually depends on multiple complementary risk factors, including host determinants (such as immune effectors), organ determinants that favor BKV replication, or external modulators (such as immunosuppressive drugs) (2). BKV is responsible for clinical manifestations in, mainly, renal-transplant patients, and rarely in heart- or liver-transplant patients, suggesting a role for local factors, such as microinflammation and replication of BKV in the renal epithelia. However, BKV-associated hemorrhagic cystitis has also been observed in 5–15% of allogeneic hematopoietic stem-cell transplants (1).

JCV does not induce disease in solid-organ transplants, but is responsible for progressive multifocal leukoencephalopathy (PML) in HIV patients and patients with multiple sclerosis treated with natalizumab. Despite specific cellular immunosuppression in these patients, nephropathy caused by JCV or BKV is rare, and BKV-associated PML or encephalitis is also rare. In addition, BKV has been rarely implicated in extrarenal pathologies such as pneumonia, encephalitis, hepatitis, retinitis, capillary-leak syndrome, or cancer (1, 3). Recently, the increased use of immunosuppressors in multiple sclerosis has been associated with emerging immunocompromised-related infections. For example, Lonergan et al. have already reported a higher incidence of BKV reactivation in patients treated with natalizumab for multiple sclerosis. However, the significance of BKV replication in the absence of renal dysfunction is unclear (6).

### Bone-marrow transplant recipients

In clinical studies, hemorrhagic cystitis caused by BKV has been observed in bone-marrow transplant recipients, mostly in young people. It can be effectively treated with a specific antiviral therapy such as cidofovir, leflunomide, or fluoroquinolone. However, controlled clinical trials with cidofovir, leflunomide, and fluoroquinolones used to treat BKV-associated hemorrhagic cystitis are needed to determine their true safety and efficacy in this patient population (7). In this population, the potential nephrotoxicity of cidofovir has caused a small number of acute renal failures.

### Renal-transplant recipients

In renal transplantation, BKV reactivation has emerged as a major complication (in 1–10% of cases) (1). Its physiopathology is still poorly understood. The first case of BKV-associated nephropathy was described in 1995 and was associated with the development of immunosuppressive drugs such as tacrolimus and mycophenolate mofetil (8). Currently, BKV remains an important issue in renal transplantation and is a major cause of infectious disease that can lead to graft loss (9, 10). BKV infections usually correspond to viral reactivation from the urinary epithelium. These reactivations occur in 1–10% of kidney-transplant (KT) recipients, and are responsible for graft loss in 30–80% of cases (1). The loss is caused on one hand by the toxic effects of the virus that cause desquamation of dying epithelial cell in urine with alteration of their nuclei (viral inclusions), and induce an inflammatory interstitial response which infiltrates tubular interstitium. This is associated with the development of fibrosis and tubular atrophy. On the other hand when immunosuppression is decreased to control the viral replication, the development of an acute tubular rejection may occur. To date, the mechanisms involved in the development of the inflammation of the kidney and the fibrosis associated with BKV nephropathy are poorly understood. The phenotype of infiltrating cells during BKV nephropathy and during acute rejection is not different in re-enforcing the difficulty to diagnose BKV nephropathy from acute rejection. Moreover, these two situations may occurred sequentially since the diagnosis of BKV nephropathy will lead to a reduction of the general immunosuppression since BKV would reflect overimmunosuppression and then will favor the development of an acute rejection which would need to re-enforce immunosuppressive therapy. This highlights the need to develop specific markers for the specific responses to this virus, to avoid episodes of acute rejection, and to control viral infection. Interestingly, the recurrence of BKV in the KT population probably needs additional factors or injured tissue to replicate. Despite its high rate of prevalence in this population, only a fraction of KT recipients will develop viral reactivation and BKV nephropathy. On the other hand, patients receiving a similar immunosuppressive therapy after heart transplantation less frequently develop BKV infection. In addition, despite an important immunosuppression, some cases of BKV have been identified in recipients of bone-marrow or cardiac transplants.

Because data are lacking on the pathophysiology of this viral reactivation, therapeutic approaches have not been codified. Currently, no antiviral treatment is available except for cidofovir, which can cause renal toxicity, particularly in those with a renal transplant (11, 12). In our experience, despite reducing the dose of immunosuppression, the use of cidofovir adapted to renal function is associated with a high rate of acute tubular necrosis.

Diagnosis of BKV-associated nephropathy is based on a combination of non-specific histological lesions plus the presence of BKV viremia (plasma BKV-DNA with a viral load of >10^4^ copies/mL), or BKV viruria (urinary BKV-DNA with a viral load >10^7^ copies/mL). Viruria is observed in 40% of KT recipients and usually precedes viremia (24% of cases) (13). Currently, because of the poor outcome from BKV nephropathy, physicians modulate immunosuppression before the occurrence of BKV nephropathy by reducing immunosuppression. Early diagnosis is essential to initiate treatment before the establishment of irreversible kidney damage (14).

**Figure 1:**
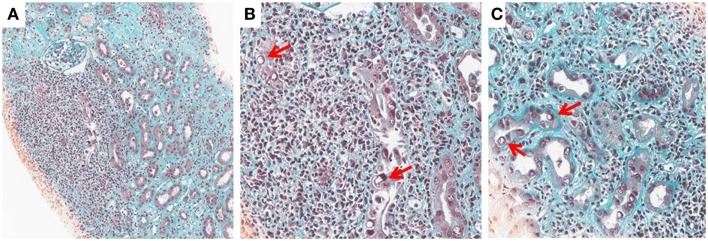
**BK-virus-associated nephropathy (A–C): intranuclear viral inclusion in epithelial cells (B,C)**. Histological analysis of a kidney section from a patient with BKV nephropathy (Trichrome Masson staining). At low magnification (x10), note an extensive fibrosis (green staining) with an important inflammation (Nucleus of leukocytes appeared in black). At higher magnification (x25), the nucleus of some tubular cells is modified by the presence of a large intranuclear viral inclusion which appeared in white (see arrows). Interstitial infiltration is made by several mononuclear cells.

Histological diagnosis is based on detecting signs of viral replication in epithelial cells (renal tubular cells and/or Bowman’s capsular cells), and/or urinary-tract (“decoy cells”). These signs include enlarged nuclei with smudgy chromatin changes, intranuclear viral inclusions, rounding, and detachment, which are associated with an inflammatory infiltrate, necrosis, and/or fibrosis. The infiltrate is mostly composed by mononuclear cells including CD4 and CD8 lymphocytes and macrophages. The virus may be identified by immunohistochemistry by staining for the Large-SV40 tumor-antigen. The following patterns of BKV-associated nephropathy have been described (1, 2, 11) (Figures 1A–C).

- Stage A – Moderate viral cytopathic changes within normal renal parenchyma.
- Stage B – More severe cellular damages with a combination of viral cytopathic changes and focal/multifocal areas of tubular atrophy, and/or interstitial fibrosis; inflammatory infiltrates, and/or tubulitis (<25% for pattern B1; 25–50% for pattern B2; >50% for pattern B3). The most important differential diagnosis is acute rejection, which can also coexist with BKV-associated nephropathy.
- Stage C – End-stage BKV-associated nephropathy, with extensive interstitial fibrosis and tubular atrophy.

According to the histological lesions, the rate of graft loss varies from 10% in stage A to 80% in stage C (1). Hirsch et al. (13) analyzed the replication of BKV in more than 600 *de novo* KT recipients within their first year post-transplant: viruria rate was 39.5% and viremia rate was 23.9%. Renal function at 1 year in viremic patients was significantly impaired (median glomerular-filtration rate 60.4 vs. 65.7 mL/min; *p* = 0.032) with more frequent acute-rejection episodes at 6 months (13.0% vs. 6.1%, *p* = 0.030). Risk factors for developing BKV viremia and/or BKV-associated nephropathy have been identified and correspond to high exposure to corticosteroids associated with tacrolimus and/or mycophenolate mofetil (compared to a lower incidence with cyclosporine A plus mycophenolate mofetil), being older, and male gender (13, 15).

The intensity of immunosuppression is conventionally proposed as a major determinant for the emergence of BKV-associated nephropathy. However, questions regarding the specific effects of immunosuppression (i.e., a direct effect or individual susceptibility) remain unanswered (13). On the one hand, patients with a conventional immunosuppressive therapy (triple immunosuppression without depleting induction with antibodies) may develop BKV replication and nephropathy, and on the other hand, patients highly immunocompromised (with depleting agents as an induction therapy for the graft or treated with high dose of steroids for the acute rejection treatment) did not. This suggests that individual susceptibility of patients might favor or control BKV reactivation.

Elfadawy et al. (16) evaluated the impact of persistent or transient BKV viremia on renal-graft function. The mean onset of BKV viremia was 4.03 ± 2.5 months post-transplant. The occurrence of BKV-associated nephropathy was limited to patients with persistent high BKV viremia. At 1 year, graft function was significantly reduced and the rate of acute rejection was significantly higher in KT recipients with high persistent or transient viremia. In addition, patients with persistent low viremia were 3.1-fold more likely to be cleared of the virus compared to those with persistent high viremia (hazard ratio = 3.1; *p* = 0.001), which suggests that potential control of the virus by the host’s immune system is associated with a low BKV viral load. This finding suggests that mechanisms other than direct tissue invasion by BKV may be responsible for graft dysfunction as well as permissive factors or mechanisms that favor the clearance of the virus and/or activation of the immune system might participate to the graft dysfunction.

### Human immunodeficiency virus 1 infected patients

Human immunodeficiency virus-1 infection is characterized by severe acquired defects in the cellular immune system, which are frequently more profound than those observed in transplant patients in the absence of a specific antiretroviral therapy. However, very rare cases of BKV-associated nephropathy have been observed. In contrast, although BKV reactivation is rare, these patients sometimes have reactivation of JCV in the brain. JCV is then responsible for PML, a rare demyelinating disease of the central nervous system that occurs almost exclusively in patients with severe defects in their cellular immune system. Incidence rates have decreased with the development of highly active antiretroviral therapies (HAART): i.e., 3.3 [95% CI, 1.9–5.7] and 1.3 [95% CI, 0.8–1.9] cases per 1000 person-years were at risk in 1995–1996 and in 2000–2006, respectively. However, survival after PML remains poor (median survival time was 0.4 years [95% CI, 0.0–0.7] before HAART and 1.8 years [95% CI, 0.6–3.0] after HAART), with ~50% mortality within 1 year of diagnosis (17) To date, there is no specific treatment for PML or a control for JCV replication. However, HAART has improved the prognosis for PML, probably by facilitating restoration of the immune defect. Indeed, after HAART initiation, an expansion of central memory CD4^+^ T cells was observed in treatment–naive HIV-infected patients and might be an early indicator of immune reconstitution (18).

## Description of the BK-Virus

Members of polyomavirus family have a common morphology and structure. Virions are small, non-enveloped, and appear as icosahedral particles 40–45 μm in diameter. Capsids contain circular double-stranded DNA with a 5 kb viral genome, wrapped around the host’s cell-derived histones. Polyomavirus genomes are more than 80% homologous. Their viral genome is divided into three regions (2, 3):

- The non-coding control region regulates gene transcription. At the origin of viral replication, this region regulates expression of the early and late viral genes.
- The early gene region encodes for regulatory proteins called the “large tumor antigen” (LTag) and “small T-antigen” (sTag). LTag and sTag facilitate viral-genome integration and replication by abrogating cell-cycle control. LTag is capable of inactivating some proteins in the endoplasmic reticulum, in particular the p53 protein, responsible for cell-cycle control by inducing apoptosis. BKV is able to prevent lysis of infected cells and has the capacity to initiate oncogenic transformation.
- The late gene region encodes the capsid proteins: VP-1, VP-2, and VP-3.

## Polyomavirus-Specific Immunity

### In healthy people

A virus-specific cellular-immunity response is critical to control virus replication and to prevent chronic disease. Viral infections with a propensity for latency require continuous immune control to restrict the rate and level of virus reactivation. T cells respond by their cytotoxic activities and the secretion of cytokines, and have a direct antiviral effect that is essential for the control of chronic viruses (19). In addition, antibodies (developed during infection) may participate in the clearance of the virus. BKV-seropositive recipients have neutralizing antibody titers, but these are not protective against BKV replication or BKV-associated nephropathy (20, 21).

The targets for BKV-specific cellular immunity are wide and T cells respond against different BKV antigens, such as VP1, VP2, VP3, LTag, and sTag. No immunodominant antigen has been identified (22, 23). The T-cell response of healthy BKV seropositives individuals has been studied by ELISpot and flow cytometry assays. Of the total tested, 75% had more than 10 spot-forming units per 10^6^ PBMC to at least one of the BKV-derived peptide pools added in the assay. Positive responses were found to peptides derived from all five major BKV proteins (VP1, VP2, VP3, LTag, and sTag). In addition, 91% of individuals had a CD4^+^ mediated response, whereas only 33% generated a CD8^+^ T-cell response (24).

BK-virus-specific cellular and humoral immunity in 122 immunocompetent individuals has been shown to be highly prevalent in young individuals. BKV-specific cellular and humoral immunity reaches its maximum in those aged between 20 and 30 years, and then declines with increasing temporal distance from the BKV primary infection. A cellular immune response is dominated by poly functional CD4^+^ T cells, which have proliferative activity and predominantly express poly functional cytokines (25).

### Polyomavirus BK in immunosuppressed patients

Episodes of BKV reactivation have a complex and poorly understood pathophysiology. Multiple factors have been suggested, such as host determinants (e.g., age, BKV-sero-negativity at pre-transplantation, specific cellular immune responses, …) and infected tissue (e.g., permissiveness for BKV replication), virus determinants (e.g., replicative characteristics), and exterior modulators (e.g., immunosuppressive treatment, co-infections, inflammatory mediators, …) (2).

In kidney transplantation, alteration of the BKV-specific T-cell response plays a crucial role in the initiation and progression of nephropathy, although we do not know the precise pathophysiological mechanisms (26, 27). Assessment of BKV-specific cellular immunity can be used in immunological monitoring to manage BKV replication. Failure to develop or expand a specific cellular immune response is a central event for the initiation and maintenance of BKV replication and the progression of BKV-associated nephropathy. BKV-specific interferon (IFN) γ-secreting T lymphocytes have been detected in the peripheral blood of healthy seropositive individuals. On the contrary, BKV-seropositive KT recipients treated with immunosuppressors had significantly lower mean frequencies of specific IFN γ-secreting T lymphocytes compared to controls, whereas no BKV-specific IFN-γ-secreting T cells were detectable in KT patients with BKV-associated nephropathy.

Reduction of immunosuppression in patients with BKV-associated nephropathy was associated with the emergence of IFN-γ-secreting T cells to the same level as that found in healthy controls, as well as decreased BKV loads in the plasma and urine (21).

In 42 KT recipients with BKV viremia, secretion of IFN-γ by T lymphocytes was demonstrated after stimulation with LTag and VP-1. This cellular immune response was significantly higher in patients with viral clearance compared to those with persistent or increased viremia. VP-1 preferentially stimulated CD4^+^ T cells whereas LTag preferentially stimulated CD8^+^ (23). CD4^+^ T cells that concomitantly secrete IFN-γ/TNF-α/IL-2 were found at a higher frequency in patients with rapid clearance of BKV, suggesting a protective role for BKV-specific poly functional CD4^+^ T cells (22, 28). Surprisingly, in patients with BKV-associated nephropathy, expansion of the effector memory CD4^+^ T cells has been observed. These suggest that not only the control of T-cell proliferation but those other mechanisms of differentiation may also be involved in the control/expansion of BKV. In addition, despite the relatively common use of immunosuppression, the high frequency of seroprevalence in the transplant population and the small number of patients that develop a BKV nephropathy suggest that there may be individual susceptibility to BKV replication within this population.

The quality of response to BKV has been discussed in other studies. The frequencies of BKV-specific CD4^+^ T cells were significantly higher in transplant recipients with BKV replication compared to T-cell frequencies in age-matched healthy controls. Interestingly, BKV replication in renal-transplant patients was associated with a significant change in T-cell functionality, with a lower proliferative activity and lower levels of poly functional T cells compared to healthy controls. A decrease in triple cytokine productive cells was observed with a concomitant increase in cytokine-single productive cells (25). These results suggest that the poly functionality of T cells, in response to the virus, may be important for virus control. The incidence of BKV nephropathy has been reported to be less frequent with the use of mTOR inhibitors-based therapy as compared to CNIs-based therapy. Because mTOR inhibitors impair the cell cycle and have be reported to reduce proliferation of virus *in vitro*, we can speculate that mTor inhibitors can reduce the replication of the virus. However, we cannot rule out that the use of mTor inhibitors could have a lower immunosuppressive function. For example, it has been suggested that mTOR inhibitors are associated with a higher rate of cellular acute rejection and a higher development of anti donor-specific antibodies and chronic humoral rejection in renal transplant.

Interestingly, JCV is responsible for PML in HIV immunocompromised patients who have a CD4 deficiency, whereas JCV is rarely responsible for disease in organ-transplant patients. Similar to BKV, a T-cell response seems to play a key role in controlling JCV replication. A low CD4^+^ T-cell count (≥50 cells/μL), in particular, and a naive subset with a high number of JCV DNA copies when PML is diagnosed appear to be risk factors for mortality (17, 29, 30). A critical role for JCV’s specific CD4^+^ T-cell responses to control JCV infection has been reported. The recovery of the anti-JCV T-cell response is associated with JCV clearance and improved survival. In addition, JCV-specific cytotoxic CD8^+^ T-lymphocytes (CTL) are undetectable in the active form of PML. Early detection of JCV-specific CTL had a 87% predictive value for subsequent JCV-control, whereas the absence of CTL had a 82% predictive value for subsequent active PML (*p* = 0.0009) (31).

In PML patients, JCV-specific CD8^+^ cytotoxic T-lymphocytes have been shown to overexpress Programed Cell Death-1 (PD-1), an inhibitory receptor, associated with cellular exhaustion. Binding of PD-1 to its ligands, PD-L1 and PD-L2, renders T-lymphocytes anergic, preventing proliferation and the production of interleukin-2 (IL-2). Blocking the PD-1 receptor *in vitro*, increased the JCV-specific T-cell immune response in HIV-positive patients with early PML (32). These results highlight the different mechanisms that can be involved in virus maintenance and that modulation of co-stimulation through the second signal family may play a role in the antiviral response.

## Experimental Models for Polyomavirus

To determine the role of the immune system in the control of the polyomavirus replication, several models of viral infection have been developed. Recently, a mouse model to study the JCV-specific immune response has been developed in humanized mice. These mice are able to develop T- and B-cell-specific human responses following contact with JCV. Six weeks after JCV inoculation, both CD4^+^ and CD8^+^ T cells overexpressed PD-1 that was found on CD4^+^ and CD8^+^ JVC-specific T cells in patients with PML, suggesting that JCV infection may have an effect on immune exhaustion (33).

No animal model has been yet conducted on BKV. However, rodents are natural hosts for murine polyomavirus (MPyV). MPyV has extensive sequence homology with the human polyomavirus. If the primo-infection is asymptomatic, viral reactivation occurs during episodes of immunosuppression. Mice infected with MPyV are a good model to study the pathophysiology of human polyomavirus infections (34, 35). Following renal transplantation, these mice developed an MPyV-associated nephropathy, similar to human BKV-associated nephropathy. Replication of MPyV was greatest, almost exclusively in kidney transplant, and was associated with renal graft loss, exacerbation of alloreactivity of CD8^+^ T cells, and an enhanced anti-donor T-cell response that led to rejection (36). This replication of MPyV occurred almost exclusively in kidney transplant, but not in native kidneys of this renal transplant mice model. This has raised the hypothesis that renal cells undergoing regeneration and differentiation, because of damage induced by transplantation or because of local inflammation, became permissive to the virus (36, 37).

## Immune Antiviral Therapeutic Options

### Specific immunological monitoring

Immuno-virological monitoring (by studying the functional capacity of specific T cells) can be used to identify patients at risk for infectious complications during acquired immunodeficiency (19). The identification of a low anti viral response against BKV, in kidney transplant could be a marker of a higher risk to develop BKV recurrence and/or BKV nephropathy. In such cases, the modulation of the immunosuppression during transplantation might enable expansion of BKV-specific T cells and, thus, the control of viral replication. The detection of BKV-specific T cells can be used as tool to guide immunosuppression with the aim of controlling viral replication while also maintaining adequate immunosuppression to prevent rejection (27).

### Innovative immune-based therapeutic treatment

Immune-based therapies may further contribute to BKV control and could be an alternative to the current treatment options in restoring an effective viral-specific immune response. A method for the generation of BKV-specific cytotoxic T lymphocytes from BKV seropositive healthy donors and KT patients, based on stimulation of PBMC with dendritic cells pulsed with inactivated BKV, has been described (38).

*Ex vivo* studies have shown that the generation and expansion of BKV-specific T cells were possible in KT recipients and allogeneic hematopoietic stem-cell transplant patients. These specific lymphocytes were obtained from hematopoietic stem cells from healthy donors or after *ex vivo* expansion of BKV-specific T cells. These cells had the ability to proliferate after antigen re-stimulation, to produce pro-inflammatory cytokines, and to induce cytotoxicity *in vitro* (39).

Another immune-based therapy could be developed from vaccines. Finding immunodominant peptides would be of interest in this context to develop a peptide vaccination.

In addition, identification of the role of the PD1/PDL1-L2 receptor, a member of the second signal activation family, during JCV infection opens up new perspectives. During JCV infection, PD1 expressed by infected cells impairs T-cell activation and virus control. Regulation of this pathway or inhibition of its signal could be potentially helpful and control viral replication. However, its modulation in transplant patients could be associated with wider activation of T-cell function, leading to acute rejection.

## Conflict of Interest Statement

The authors declare that the research was conducted in the absence of any commercial or financial relationships that could be construed as a potential conflict of interest.
